# Posttransurethral Resection of Prostate Recurrent Life Threatening Hematuria: A Rare Cause

**DOI:** 10.1155/2016/5895016

**Published:** 2016-04-12

**Authors:** MC Arya, Lalit Kumar, Ruchi Mittal, Rajeev Kumar, Mayank Baid

**Affiliations:** ^1^Department of Urology, Sardar Patel Medical College, B-4/10-11, Sudarshan Nagar, Bikaner, Rajasthan 334001, India; ^2^Sardar Patel Medical College, Bikaner, Rajasthan, India

## Abstract

Herein, we present a case report of post-TURP (transurethral resection of prostate) recurrent severe hematuria due to right internal iliac artery pseudoaneurysm protruding into bladder lumen. A 60-year-old male presented with recurrent massive hematuria following TURP done elsewhere 15 days before. His hemoglobin was 4 gm/dL after 13 units of blood transfusion and repeated clot evacuations. His blood urea, serum creatinine, and coagulation profile studies were normal. Ultrasonography of abdomen showed multiple clots in the bladder. Cystoscopy revealed clots with a right posterolateral wall unhealthy area. After stabilizing the patient, contrast enhanced CT urography revealed intravesical aneurysm. CT angiography showed pseudoaneurysm of a branch of internal iliac artery protruding into urinary bladder lumen. We referred patient to selective embolization of the lesion but the procedure was unsuccessful. At last, ipsilateral internal iliac artery ligation relieved hematuria. But on postoperative day 2, patient suddenly collapsed and deceased, presumably due to cardiomorbidities.

## 1. Introduction

Iliac artery aneurysms coexist with aortic aneurysms in around 10–20% of cases [1]. However, solitary internal iliac artery aneurysms (IIAA) are extremely rare, with a prevalence of around 0.3–0.4% of all intra-abdominal aneurysms [2]. Most of patients with IIAA are either asymptomatic or rarely present with abdominal pain, urinary symptoms, lumbosacral pain, hip or buttock pain, rectal bleeding, or constipation [3].

Post-transurethral resection of prostate (TURP) bleeding is not uncommon. But recurrent hematuria after TURP should raise an alert in mind. Herein, we present a case report of post-TURP recurrent severe hematuria due to right internal iliac artery aneurysm protruding into bladder lumen.

## 2. Case History

A 60-year-old man was presenting with recurrent massive hematuria following TURP done elsewhere 15 days before.

Haematological investigation at the time of presentation is as follows:

- Haemoglobin level: 4 gm/dL after 13 units of blood transfusion and repeated clot evacuations.
- Blood urea and serum creatinine level: normal.
- Coagulation profile: normal.

**Figure 1:**
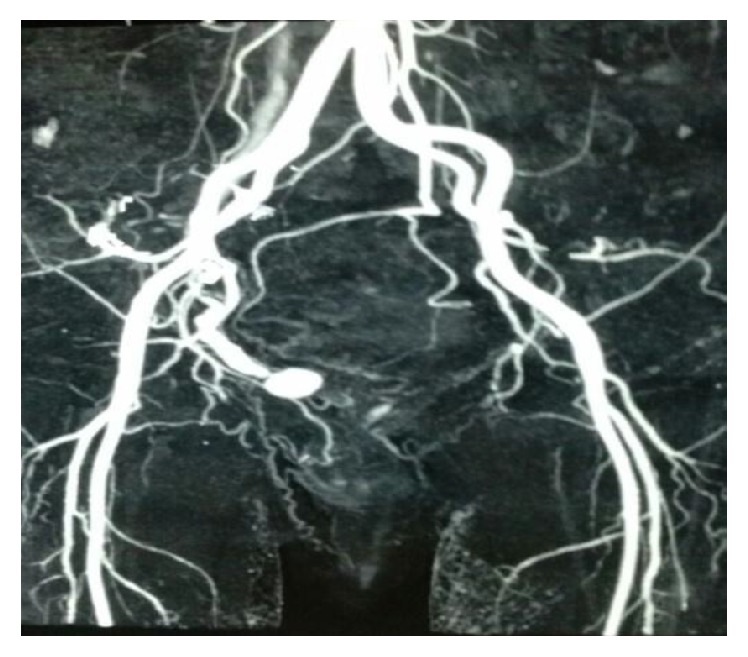
Pseudoaneurysm of branch of right internal iliac artery.

**Figure 2:**
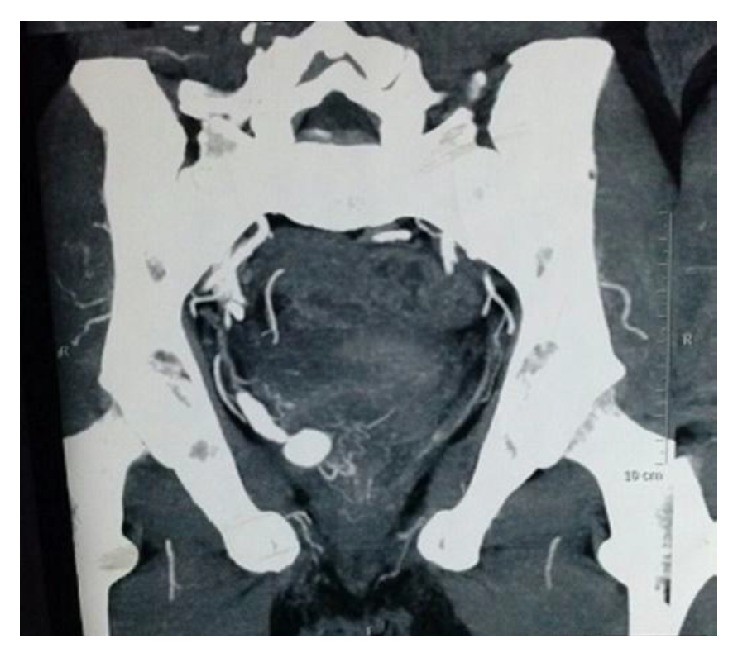
Demonstrated terminal pseudoaneurysm protruding into bladder lumen.

Diagnosis at the time of presentation is as follows:

- Ultrasonography of abdomen showed multiple clots in the bladder.
- Cystoscopy revealed clots with a right posterolateral wall unhealthy area.
- After stabilizing the patient, contrast enhanced CT urography revealed intravesical aneurysm.
- CT angiography showed fusiform aneurysmal dilatation of vesicle territory of internal iliac artery with maximum caliber up to 8.5 mm and approximately 31 mm long segment (Figure 1).
- A well defined terminal pseudoaneurysm formation, measuring approximately 13.3 × 15.3 × 11.6 mm, was protruding in urinary bladder lumen (Figure 2).

*Surgical Procedure and Outcome*. We referred the patient to cardiologist for selective embolization of the lesion. Embolization was done by puncturing left femoral artery and through left femoral artery to left external iliac artery and then to abdominal aorta and right common iliac artery, and finally right iliac artery was cannulated. 4 Fr cobra catheter was used to selectively cannulate right iliac artery branch. Single cook embolization coil (35-5-5) was used. Two days after embolization, the patient presented again with hematuria and on follow-up CT angiography lesion had not completely disappeared.

Right internal iliac artery ligation was performed below origin of superior gluteal artery to avoid claudication and hematuria subsided. On postoperative day 2, the patient suddenly collapsed and deceased. So we should be more vigilant regarding aneurysm in the bladder. In the present case, postmortem was not done. In India, it is mandatory only in medicolegal cases.

## 3. Discussion

Isolated IIA aneurysms are very rare. 25–40% of IIAA patients present with rupture [3]. These are generally confined up to the retroperitoneal space and rarely rupture into the peritoneal space presenting with shock and peritonitis. These can also rupture into adjacent viscera like rectum, bladder, and common iliac vein [4].

Internal iliac artery aneurysm or pseudoaneurysm presenting as hematuria is an uncommon entity. Up to date, in literature, only 18 cases of vesicoureteral fistula with hematuria due to varied etiologies have been reported. Nine cases presented an iatrogenic cause and 4 were due to trauma, while in 5 spontaneous rupture of aneurysm into the bladder occurred. In one case, hematuria occurred as a result of bleeding of bladder mucosa due to congestion of perivesical vessels resulting from aneurysm compression [4–10].

In the present case, the etiology was iatrogenic as the patient had a history of previous TURP done elsewhere. Bleeding and loss of vision possibly could have caused bladder trauma. He presented with recurrent hematuria with clot retention. We could not point out diagnosis thinking it as a common complication of TURP. Later diagnosis was made clear by CT urography and CT angiography. CT angiography demonstrated a pseudoaneurysm of a branch of the right internal iliac artery that was likely caused during the previous TURP. Diagnosis of a pseudoaneurysm of a vesical artery is difficult since it is a very rare entity. But we should always have this rare possibility in our mind so diagnosis can be made at early stage that will help in better and precise management.

In our patient, there was no history of hypertension or any other cardiac illness on history. Unfortunately, our patient expired due to some hidden cardiovascular pathology or pulmonary embolism. So, beside CT angiography, we should have 2D echo or evaluation for risk factors for pulmonary embolism.

Management options are either selective embolization, resection, repair, or exclusion by open approach [8]. In this case, we referred the patient to cardiologist for embolization as patient general condition was not good and it was looking like a pseudoaneurysm of a small length of artery. But embolization attempts failed and ipsilateral internal iliac artery ligation was performed as a last resort. It achieved its goal of hematuria resolution.

## 4. Conclusion

Rupture of internal iliac artery pseudoaneurysm protruding into bladder lumen presenting as a recurrent massive hematuria is very rare and thus warrants presentation. So we should be more vigilant regarding aneurysm in the bladder and should consider possibility of this rare diagnosis as a cause of macroscopic hematuria. Attempting fulguration or resection in such a case could be fatal. If we have suspicion of such lesion in bladder while doing endoscopic procedure, one should refer to CT urography or CT angiography. We should thoroughly evaluate such cases especially from cardiovascular point of view as there is high risk of sudden death from cardiac diseases or pulmonary embolism. Awareness of this as a possible cause of hematuria can assist in immediate diagnosis and appropriate treatment.

## Abbreviations

TURP: Transurethral resection of prostate
IIAA: Internal iliac artery aneurysm.
